# Supplementary material for: The Systems Biology Research Tool: evolvable open-source software
Source: BMC Syst Biol. 2008 Jun 29;2:55. doi: 10.1186/1752-0509-2-55 (PMC2446383; doi:10.1186/1752-0509-2-55)
Supplement: Additional file 1 — SBRT Archive. An archive of the current version of the Systems Biology Research Tool. [file 1752-0509-2-55-S1.zip › sbrt-1.4.0/doc/developers_guide/api/sbrt/kernel/fba/comp/SlpCycleComputer.html]

SlpCycleComputer


|  |  |  |
| --- | --- | --- |
| **Overview** \| **Package** \| **Class** \| **Use** \| **Tree** \| **Deprecated** \| **Index** \| **Help** | |  |
| **PREV CLASS**   **NEXT CLASS** | **FRAMES**    **NO FRAMES**     **All Classes** |  |
| SUMMARY: NESTED \| FIELD \| CONSTR \| METHOD | DETAIL: FIELD \| CONSTR \| METHOD |  |


---


## sbrt.kernel.fba.comp Class SlpCycleComputer

```
java.lang.Object
  sbrt.kernel.fba.comp.SlpCycleComputer
```

**All Implemented Interfaces:**: CycleComputer

---

``` public class SlpCycleComputer extends java.lang.Object implements CycleComputer ```

This class is used to identify the steady state cycles (Type III extreme
pathways) in a fluxome.
It is an implementation of the algorithm described by Schilling, Letscher,
and Palsson (SLP).

**Author:**
:   This class was written and documented by Jeremiah Wright while in the
    Wagner lab.

---

**Constructor Summary**

`SlpCycleComputer(ExtremeCurrentComputer computer)`
  
          Constructs a new steady state cycle computer from the given extreme
current computer.


| **Method Summary** | |
| --- | --- |
| `SparseDoubleMatrix<?,java.lang.String>` | `getCycles(IrrevRxnFluxome fluxome)`             Returns the set of all steady state cycles (Type III extreme pathways) contained in the provided fluxome. |
| `ProgressMonitor` | `getProgress()`             Returns a progress monitor for this steady state cycle computer. |

**Methods inherited from class java.lang.Object**

`clone, equals, finalize, getClass, hashCode, notify, notifyAll, toString, wait, wait, wait`

**Constructor Detail**

### SlpCycleComputer

```
public SlpCycleComputer(ExtremeCurrentComputer computer)
```

:   Constructs a new steady state cycle computer from the given extreme
    current computer.

    **Parameters:**: `computer` - the computer with which all cycles will be computed.


**Method Detail**

### getProgress

```
public ProgressMonitor getProgress()
```

:   Returns a progress monitor for this steady state cycle computer.

    :   **Specified by:**: `getProgress` in interface `CycleComputer`
    :   **Returns:**: a progress monitor for this steady state cycle computer.

---


### getCycles

```
public SparseDoubleMatrix<?,java.lang.String> getCycles(IrrevRxnFluxome fluxome)
```

:   Returns the set of all steady state cycles (Type III extreme pathways)
    contained in the provided fluxome.

    :   **Specified by:**: `getCycles` in interface `CycleComputer`
    :   **Parameters:**: `fluxome` - the fluxome for which all cycles will be identified. **Returns:**: the extreme current matrix. Column variables are (all) internal reaction names, and each row represents a steady state cycle.


---


|  |  |  |
| --- | --- | --- |
| **Overview** \| **Package** \| **Class** \| **Use** \| **Tree** \| **Deprecated** \| **Index** \| **Help** | |  |
| **PREV CLASS**   **NEXT CLASS** | **FRAMES**    **NO FRAMES**     **All Classes** |  |
| SUMMARY: NESTED \| FIELD \| CONSTR \| METHOD | DETAIL: FIELD \| CONSTR \| METHOD |  |


---
